# Serum Carcinoembryonic Antigen Is Associated with Abdominal Visceral Fat Accumulation in Female Korean Nonsmokers

**DOI:** 10.1371/journal.pone.0043518

**Published:** 2012-08-27

**Authors:** Jee-Yon Lee, Hyang-Kyu Lee, Duk-Chul Lee, Ji-Won Lee

**Affiliations:** 1 Department of Family Medicine, Severance hospital, Yonsei University, College of Medicine, Seodaemun-gu, Korea; 2 Department of Clinical Nursing Science, College of Nursing, Yonsei, University, Seodaemun-gu, Korea; College of Tropical Agriculture and Human Resources, University of Hawaii, United States of America

## Abstract

**Background:**

Carcinoembryonic antigen (CEA) is a tumor marker overexpressed in adenocarcinoma that has proinflammatory properties. Recent studies have reported that CEA is positively associated with carotid atherosclerosis and metabolic syndrome. Because visceral obesity is a known risk factor for cardiometabolic diseases, CEA may also be associated with visceral adiposity. Therefore, we investigated the relationship between serum CEA concentration and visceral obesity in female Korean nonsmokers.

**Methods:**

A total of 270 Korean female nonsmokers were enrolled during their routine health check-ups. Biomarkers of metabolic risk factors were assessed along with body composition by computed tomography. Serum CEA levels were measured by using a chemiluminescence immunoassay analyzer.

**Results:**

Serum CEA levels correlated with visceral fat area, fasting glucose, and triglyceride levels after adjusting for age and BMI. The mean visceral fat area increased significantly with the increasing CEA tirtiles. In a step-wise multiple regression analysis, age (*β = *0.26, p<0.01) and visceral fat area (*β = *0.19, p = 0.03) were identified as explanatory variables for serum CEA level.

**Conclusions:**

This study suggested that CEA may be a mediator that links metabolic disturbance and tumorigenesis in visceral obesity. Further studies are required to better understand the clinical and pathophysiological significance of our findings.

## Introduction

Obesity is a risk factor for cardiovascular disease [1], diabetes mellitus [1], and neoplastic conditions [2]. Previous studies have reported that obesity is positively associated with mortality due to cardiovascular disease, diabetes mellitus, and certain cancers [3], [4]. The precise mechanism by which obesity promotes these health problems is not entirely clear; however, regional distribution of adipose tissue is thought to be a contributing factor [5]. Visceral adipose tissue is more metabolically active than subcutaneous adipose tissue [6] and has a stronger relationship with cardiometabolic risk factors including dyslipidemia [7], hyperinsulinemia [8], and tumorigenesis. Although the role of visceral adipose tissue in these diseases has not been fully elucidated, visceral adipocytes secrete cytokines, growth factors, and adhesion molecules that promote the development of obesity-related pathologic conditions [9].

Carcinoembryonic antigen (CEA) is a 180- to 200-kDa glycoprotein (10) that is a widely used tumor marker [10]. CEA is overexpressed in adenocarcinoma, especially colorectal cancer [11]. Its efficacy in monitoring recurrence after colorectal cancer treatment has been demonstrated in numerous studies [12]. However, CEA is not considered effective as a marker for cancer screening, because CEA levels increase with age and are elevated in many nonneoplastic conditions including smoking, inflammatory bowel disease, and chronic hepatitis [13]–[15]. Furthermore, recent studies have reported a positive association between CEA and cardiometabolic diseases including carotid atherosclerosis [16] and metabolic syndrome [17]. Although CEA stimulates production of proinflammatory cytokines and adhesion molecules during the development of atherosclerosis, insulin resistance (IR), tumorigenesis, and metastasis, the precise mechanism underlying the relationship between CEA and cardiometabolic diseases remains unclear.

Because visceral obesity is known to be a major risk factor for atherosclerosis, IR, and malignancy, CEA may be associated with visceral adiposity. However, no studies have evaluated this relationship. Therefore we investigated the relationship between serum CEA concentration and visceral obesity in female Korean nonsmokers.

## Methods

### Ethics Statement

All subjects participated in the study voluntarily, and written informed consent was obtained from each participant. The study complied with the Declaration of Helsinki and the institutional review board of Yonsei University College of Medicine approved this study.

### Study sample

**Figure 1:**
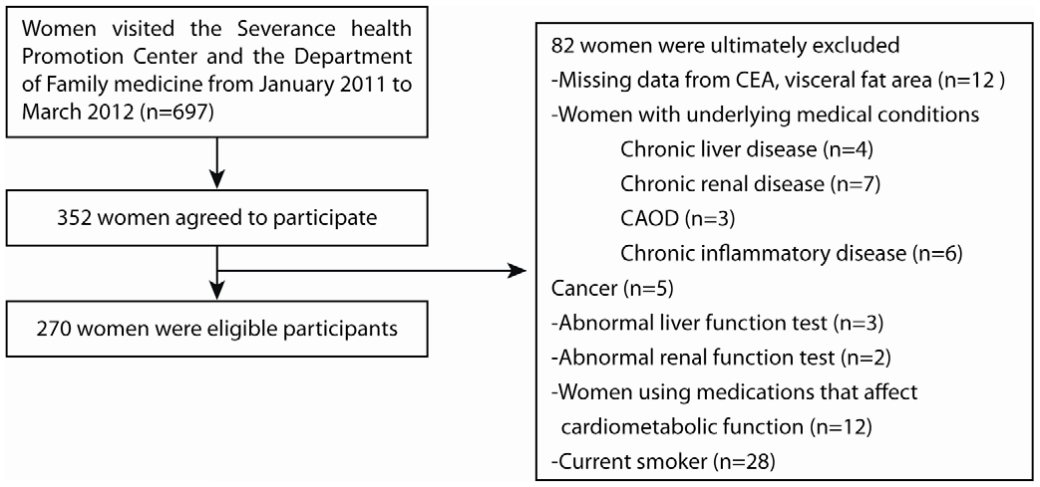
Patient selection flow-chart for our study evaluating the association of CEA levels and visceral obesity.

The study sample consisted of 352 Korean women who visited the Health Promotion Center and the Department of Family Medicine at Severance Hospital for routine health check-ups between January 2011 and March 2012. From the 697 women, a total of 352 women agreed to participate in the study, so the initial participation rate was 50.5%. Of the 352 women who were initially enrolled, 270 women met all inclusion criteria and were ultimately included in the study analysis, while 82 women were excluded from further analysis after medical history screening. We excluded 12 women who were missing data for CEA concentrations and abdominal visceral fat area, and 28 women who were current or former smokers. We also excluded women with underlying medical condition including a history of chronic liver disease (n = 4), chronic renal disease (n = 7), coronary artery occlusive disease (n = 3), chronic inflammatory disease (e.g., pancreatitis, COPD; n = 6) or cancer (n = 5). Women with abnormal liver (n = 3) or kidney function (n = 2) were also excluded. Abnormal liver function was defined as serum aspartate aminotransferase or alanine aminotransferase concentrations >100 IU/L. Abnormal kidney function was defined as serum creatinine concentration >1.4 mg/dL. We also excluded women using any medications that could affect cardiometabolic function including anti-hypertensive medicine, oral hypoglycemic agents, lipid lowering agents, anti-obesity drugs, and antidepressants.(n = 12; Figure 1). A total of 270 women were included for the final analysis.

### Measurements

All subjects completed a questionnaire about lifestyle factors, including cigarette smoking and alcohol consumption (defined as drinking alcohol more frequently than once a week). Anthropometric measurements were taken by a single well-trained examiner. Blood pressure was measured in the sitting position after a 10-minute resting period. Body mass index was calculated as weight divided by height squared. Waist circumference was measured at the umbilicus while the subject was standing. Bioelectrical impedance analysis was used to estimate body fat percentage using the InBody 520 (Biospace, Seoul, Korea). Abdominal fat tissue areas were measured by computed tomography (Tomoscan 350; Philips, Mahwah, NJ, USA) as described previously [18]. Blood samples were collected after a 12-hour overnight fast. We measured fasting glucose, high-sensitivity C-reactive protein (hs-CRP), total cholesterol, triglyceride, and high-density lipoprotein (HDL)-cholesterol levels by using an ADVIA 1650 chemistry system (Siemens Medical Solution, Tarrytown, NY, USA). Low-density lipoprotein (LDL)-cholesterol levels were calculated using the Friedewald equation. Fasting insulin was determined by electrochemiluminescence immunoassay using an Elecsys 2010 (Roche, Indianapolis, IN, USA). IR was estimated by the homeostasis model assessment of insulin resistance (HOMA-IR) index: (insulin [µIU/mL] × fasting blood glucose [mg/dL]/18)/22.5.

CEA was measured by a chemiluminescence immunoassay (CLIA) using a DxI 800 Access Immunoassay System (Beckman Coulter Inc., Brea, CA, USA). Assay precision was evaluated by testing three control concentrations of commercially available quality control materials (MAS T-Marker; Medical Analysis Systems, Camarillo, CA, USA) and pooled human serum for the respective markers. Each sample was assayed twice, in duplicate each time, with a minimum of 2 hour intervals between runs. The inter-assay and intra-assay coefficients of variations were 3.80±1.96% and 3.01±1.55% respectively.

The hypertension group was defined as subjects with systolic BP≥140 mmHg or diastolic BP≥90 mmHg. The diabetes group was defined as subjects with fasting blood glucose ≥126 mg/dL. The dyslipidemia group was defined as subjects with hypercholesterolemia (>240 mg/dL), hypertriglyceridemia (>150 mg/dL), or low HDL-cholesterol (<50 mg/dL).

### Statistical analysis

Normally distributed data are expressed as mean ± standard deviation (SD), and non-normally distributed data are expressed as median and interquartile range. Pearson and Spearman correlation analysis was performed to evaluate relationships between CEA and other metabolic variables. The mean visceral fat area of the participants according to the CEA tertiles was compared using one-way ANOVA. Multiple linear regression analysis was used to identify factors contributing to serum CEA levels. For this analysis, variables with p<0.2 from univariate analysis and clinically important variables including age, BMI, and subcutaneous fat area were entered. To avoid multicollinearity, if there was significant correlation (r>0.7) between two variables, only one variable was selected and entered into the model.

We performed all statistical analysis using the Statistical Package for the Social Sciences, version 18.0 (SPSS Inc., Chicago, IL, USA). Statistic significance was defined as p<0.05.

## Results

Table 1 shows the clinical characteristics of the 270 subjects and the relationships between serum CEA level and other variables. The mean age of subjects was 41.46±14.0 years, and median CEA level was 1.06 ng/mL(0.68–1.58). After adjusting for age and BMI, CEA levels remained significantly correlated with visceral fat area (r = 0.17 p = 0.01), fasting glucose (r = 0.17, p = 0.01), and triglyceride levels (r = 0.13, p = 0.03). The mean CEA level was significantly higher in hypertensive group (1.63 ng/mL) versus the normotensive group (1.16 ng/mL; p<0.01), in diabetic group (1.87 ng/mL) versus the non-diabetic group (1.18 ng/mL; p<0.01) and in dyslipidemia group (1.58 ng/mL) versus the non-dyslipidemia group (1.19 ng/mL; p = 0.02). There were no significant differences in mean CEA levels between the alcohol group 0.88 ng/mL and the non-alcohol group (1.25 ng/mL; p = 0.28). (Data not shown)

**Table 1:** Clinical characteristics of study subjects and the correlation between serum CEA levels and various metabolic parameters.

Variables	Total (n = 270)	r	p-value	Age, BMI adjusted	
				r	p
CEA (ng/mL)	1.06 (0.68–1.58)				
Age (years)	41.46±14.00	0.43	<0.01		
Adiposity index					
BMI (kg/m^2)^	27.22±5.08	0.02	0.72		
Waist (cm)	89.72±9.58	0.06	0.33	0.04	0.48
Body fat (%)	36.47±6.71	−0.01	0.93	0.01	0.83
Visceral fat area (cm^2)^	98.79±49.44	0.25	<0.01	0.17	0.01
Subcutaneous fat area (cm^2^)	263.00±92.66	−0.02	0.75	0.00	0.99
Blood pressure (mmHg)					
Systolic	120.00 (113.00–131.75)	0.11	0.08	0.02	0.78
Diastolic	74.26±9.50	0.22	<0.01	0.06	0.33
Fasting glucose (mg/dL)	91.00 (86.00–98.00)	0.24	0.01	0.17	0.01
Fasting insulin (µIU/mL)	8.17 (5.50–12.97)	0.13	0.03	0.05	0.44
HOMA-IR	1.88 (1.23–2.97)	−0.07	0.26	0.09	0.16
Lipid profile (mg/dL)					
Total cholesterol	187.56±35.27	0.09	0.16	0.05	0.44
Triglyceride	92.50 (64.75–137.25)	0.18	<0.01	0.13	0.03
HDL-cholesterol	52.58±13.21	−0.07	0.29	−0.01	0.87
LDL-cholesterol	112.84±31.64	0.05	0.45	0.00	0.93
hs-CRP (mg/L)	0.86 (0.48–1.88)	0.04	0.61	−0.01	0.93
Hypertension, n (%)	50 (18.30%)				
Diabetes, n (%)	22 (8.20%)				
Dyslipidemia, n (%)	29 (10.60%)				
Alcohol drinking, n (%)	10 (3.70%)				

Note: BMI, body mass index; HOMA-IR; homeostasis model of assessment of insulin resistance; HDL, high-density lipoprotein; LDL, low-density lipoprotein; hs-CRP, high-sensitivity C-reactive protein.

Alcohol consumption was defined as consuming alcohol more frequently than once a week.

Data are expressed as mean (±SD) or percentage. Skewed data are expressed as median (range).

Coefficients(r) and p-values were calculated by the Pearson correlation model (normally distributed variables: age, BMI, waist circumference, subcutaneous fat area, diastolic blood pressure, fasting glucose, total cholesterol, and LDL-cholesterol) or Spearman correlation model (non-normally distributed variables: body fat, visceral fat area, systolic blood pressure, fasting insulin, HOMA-IR, triglycerides, HDL-cholesterol, and hs-CRP).

**Figure 2:**
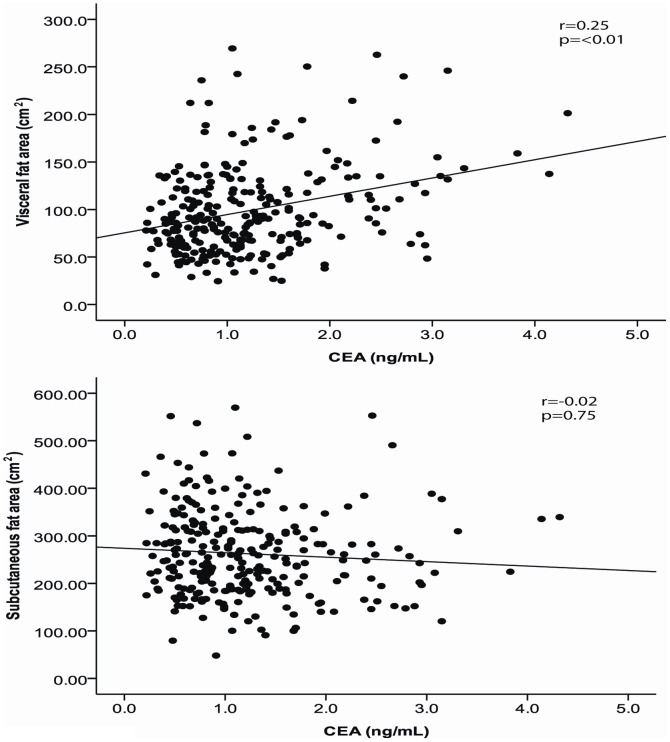
Relationship between abdominal visceral fat area, abdominal subcutaneous fat area, and serum CEA levels.

**Figure 3:**
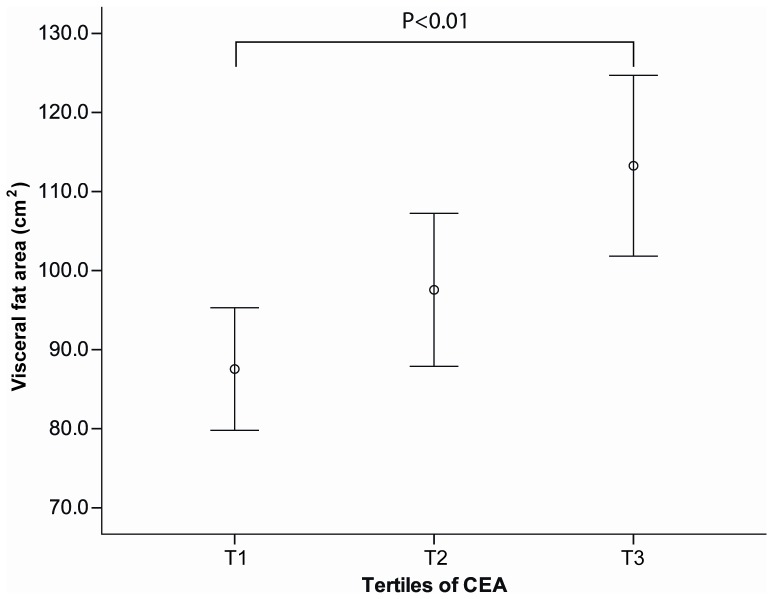
Abdominal visceral fat area according to the CEA tertile in female Korean non-smokers. P-values were calculated using one-way analysis of variance. Bars indicate 95% confidence intervals.

Figure 2 and 3 shows the relationship between CEA and abdominal adiposity. Serum CEA level correlated positively with visceral fat area but not with subcutaneous fat area (Figure 2). Figure 3 shows the mean visceral fat area according to the CEA tertiles. (T1, <0.9; T2, 0.9–1.4; T3> = 1.4 ng/ml) Visceral fat area increased continuously according to the CEA tertiles.

In a linear multiple regression analysis, age (*β = *0.26, p<0.01) and visceral fat area (*β = *0.19, p = 0.03) were identified as significant explanatory variables for serum CEA levels, accounting for a combined 22% of the variance in CEA concentration. (Table 2) The relationship between CEA level and visceral fat area remained significant after exclusion of participants with hypertension (n = 50) and diabetes (n = 22) (*β* = 0.36, r<0.01).

**Table 2:** Multivariate linear regression analysis to determine relationships between CEA levels and clinical metabolic variables.

Variable	*β* coefficient	SE	p
Age (years)	0.26	0.01	<0.01
Systolic blood pressure (mmHg)	−0.04	0.01	0.61
Diastolic blood pressure (mmHg)	0.02	0.01	0.84
BMI (kg/m^2^)	0.05	0.02	0.59
Visceral fat area (cm^2^)	0.19	0.002	0.03
Subcutaneous fat area (cm^2^)	−0.04	0.001	0.65
Fasting glucose (mg/dL)	0.11	0.003	0.08
Fasting insulin (µIU/mL)	0.01	0.01	0.90
Total cholesterol (mg/dL)	0.01	0.001	0.92
Triglyceride (mg/dL)	0.08	0.001	0.33
HDL-cholesterol (mg/dL)	0.05	0.004	0.44

R^2^ = 0.22. All variables left in the model are significant at the 0.15 level. No other variable met the 0.15 significance level for entry into the model. Variables included in the model for CEA: age, BMI, visceral fat area, subcutaneous fat area, systolic blood pressure, diastolic blood pressure, fasting glucose, fasting insulin, total cholesterol, triglycerides, HDL-cholesterol.

## Discussion

Our cross-sectional study revealed a relationship between serum CEA level and visceral obesity in female Korean nonsmokers. This association remained significant after adjusting for BMI as an overall marker of obesity, and for other confounding factors related to metabolic syndrome including glucose, insulin, total cholesterol, triglycerides, HDL-cholesterol, and blood pressure.

CEA is used to monitor disease recurrence and therapeutic efficacy in colorectal cancer [19]. However, serum CEA levels are also mildly elevated in several nonmalignant conditions [13]–[15], including metabolic disturbances. Furthermore, Nobukarzu et al [16] reported a positive relationship between carotid atherosclerosis and serum CEA concentration. In our previous studies [17], serum CEA was positively associated with metabolic syndrome in a concentration-dependent manner. However, the relationship between CEA and fat distribution is yet not well understood. This is the first study to evaluate the relationship between serum CEA levels and abdominal visceral obesity.

The precise underlying mechanisms that explain the relationship between serum CEA concentration and visceral obesity remain unclear. To our knowledge, there are no prior experimental studies that evaluated the role of CEA in visceral adiposity. Whether CEA levels and visceral fat amounts are directly associated, or if this relationship is mediated by other environmental factors, has not yet been figured out. Therefore, additional basic experimental studies are warranted. However, we propose two possible mechanisms.

First, if the association between CEA and visceral fat is mediated by environmental factors, then inflammatory cytokines and adipokines could be possible intermediary candidates. Adipose tissues secrete various inflammatory cytokines and adipokines that induce the systemic chronic low-grade inflammatory status observed in visceral obesity [20]. Altered adipokines and cytokines in visceral obesity contribute to the development of IR, cardio-metabolic disease, and malignancy [21]–[24]. Since CEA levels are associated with diverse chronic inflammatory diseases [13], [15], increased inflammatory cytokines and adipokines in visceral obesity may stimulate the cellular expression of CEA.

Second, direct association of CEA with visceral adiposity without any mediators should be considered. It has been reported that CEA can be secreted from non-CEA producing cells under certain conditions [25], [26]. Hence, we can hypothesize that alteration of cellular environment in visceral obesity may induce the direct expression of CEA from adipocytes. Basic experimental studies are needed to elucidate the precise mechanistic association between CEA and visceral obesity.

This study has several limitations. First, the cross-sectional design cannot establish a causal relationship between CEA levels and visceral obesity and also does not exclude the possibility that CEA levels were elevated prior to visceral obesity. Second, we enrolled only females; therefore, our results may not be generalizable to men. Third, the small sample size of our study is another limitation. Fourth, we used nonprobability sampling so our results do not allow generalization of the results to the population at large. Finally, although we considered a large number of potentially confounding metabolic factors in our study, evaluation of all potential confounding factors is impossible. We agree that consideration of other potential confounders, including assessing inflammatory cytokine and adipokine levels in study subjects, will provide additional important information in future studies.

In conclusion, our study showed that serum CEA is associated with abdominal visceral obesity in female Korean nonsmokers. Our findings collectively suggest that CEA may be a mediator that links metabolic disturbance and tumorigenesis in visceral obesity. In addition, elevation CEA, even within the “normal” range, may be considered a detective marker for metabolic diseases related to visceral obesity. Further studies are required to better understand the clinical and pathophysiological significance of our findings.
